# Natural History of Primary Retroperitoneal Extra-Visceral Perivascular Epithelioid Cell Tumors (PEC): A Study from Transatlantic and Australasian Retroperitoneal Sarcoma Working Group (TARPSWG)

**DOI:** 10.1245/s10434-025-17787-8

**Published:** 2025-07-14

**Authors:** Eyal Mor, Sameer Apte, Catherine Mitchell, Carolyn Nessim, Max Almond, Bruno Vincenzi, Jose Antonio Gonzalez Lopez, Lee Cranmer, Michael J. Wagner, Aviram Nissan, Miguel Henriques Abreu, Markus Albertsmeier, Mathilda Knoblauch, Adam Barlow, Emily Z. Keung, Giovanni Grignani, Jason L. Hornick, Alessandro Gronchi, David E. Gyorki

**Affiliations:** 1 Sir Peter MacCallum Department of Oncology, Peter MacCallum Cancer Centre, University of Melbourne, Melbourne, VIC Australia; 2 University of Ottawa, Ottawa, Canada; 3 University Hospitals Birmingham NHS Foundation Trust, Birmingham, UK; 4 Università Campus Bio-Medico, Rome, Italy; 5 Hospital de Sant Pau, Barcelona, Spain; 6 University of Washington, Seattle, WA USA; 7 Dana-Farber Cancer Institute, Boston, MA USA; 8 Sheba Medical Center, Ramat-Gan, Israel; 9 Portuguese Institute of Oncology of Porto, Porto, Portugal; 10 Ludwig-Maximilians-Universität (LMU) Munich, LMU University Hospital, Munich, Germany; 11 Leeds Teaching Hospitals NHS Trust, Leeds, UK; 12 The University of Texas MD Anderson, Houston, TX USA; 13 Candiolo Cancer Institute, FPO-IRCCS, Candiolo, TO Italy; 14 Brigham and Women’s Hospital, Harvard Medical School, Boston, MA USA; 15 Fondazione IRCCS Istituto Nazionale dei Tumori, Milan, Italy; 16 Division of Cancer Surgery, Peter MacCallum Cancer Centre, Melbourne, Victoria Australia

**Keywords:** PEComa, Retroperitoneal sarcoma, Surgical oncology, Perivascular epithelioid cell tumors

## Abstract

**Background:**

Perivascular epithelioid cell tumors (PEComa) are a rare family of mesenchymal tumors that include several subtypes. There are very limited data describing the natural history of patients with extra-visceral retroperitoneal PEComas of the retroperitoneum. The aim of this study is to describe the clinical features, treatment patterns, outcomes, and diagnostic challenges of primary extra-visceral retroperitoneal or abdominopelvic PEComa over the past decade.

**Patients and Methods:**

This is a retrospective analysis of all extra-visceral, non-renal, retroperitoneal, or abdominopelvic PEComas treated at participating centers over the past 10 years.

**Results:**

A total of 77 patients from 13 centers were included. The median age at diagnosis was 56 years (range 18–81 years); 73% were female. The median size was 9 cm. The tumor was classified as a PEComa not otherwise specified (NOS) in 55 (71%), sclerosing PEComa in 11 (15%), and angiomyolipoma (AML) in 11 (15%). Treatment intent was curative in 59 (77%) patients. Adjuvant radiation was given in five (8%) patients, and (neo)adjuvant systemic therapy was given to six (10%). Of those who did not undergo curative intent treatment, four (22%) patients had metastatic disease and three (17%) had primary unresectable disease. With a median follow-up of 26 months (2.3–147 months), 24 (40%) of the 59 patients having curative treatment had recurred. Recurrence rates differed by subtype, with 20 (37%) of the PEComa NOS group, 3 (27%) of the sclerosing PEComa group, and 1 (9%) of the AML group developing recurrence. The estimated 5-year OS of the whole cohort was 63% and 75% for the curative intent group.

**Conclusions:**

Retroperitoneal and abdominopelvic PEComas show distinct behaviors by subtype. PEComa NOS had the highest recurrence and mortality, sclerosing PEComa showed intermediate risk, and AML was indolent. Histological classification is essential for prognosis and management.

**Supplementary Information:**

The online version contains supplementary material available at 10.1245/s10434-025-17787-8.

Perivascular epithelioid cell tumors (PEComa) is a term first used by Bonetti et al. in 1992^1,2^ to describe a rare family of mesenchymal tumors, including angiomyolipoma (AML) and clear cell “sugar” tumor of lung. PEComas characteristically express both muscle (actin/Desmin) and melanocytic (HMB45/Melan-A) markers^3^ as the hallmark of a putative progenitor, the perivascular epithelioid (PEC) cell,^4^ although these cells have no known normal human tissue counterpart. Since Bonetti’s initial description, the PEComa family of tumors has evolved to include other subtypes such as PEComa NOS, sclerosing PEComa, and lymphangioleiomyomatosis (LAM).^3,5,6^ Common sites of PEComa occurrence are the kidney, liver, lung, abdominopelvic soft tissues, gastrointestinal organs, retroperitoneum, and the skin. Due to its rarity, literature regarding primary PEComa of the retroperitoneum is restricted to case reports and small case series. Consequently, the natural history, clinicopathologic characteristics, common treatment strategies, and oncologic outcomes for extra-visceral, primary retroperitoneal PEComas is largely unknown. The literature that is available, however, implies that high mitotic rate (> 1 mm/5 mm^2^), size > 5 cm, necrosis, and infiltrative growth pattern may be associated with aggressiveness.^5,7,8^ Expert consensus is that surgical resection is the optimal primary treatment modality, however, some subtypes of PEComa may respond to mTOR inhibitors if unresectable.^9^ This study aims to compile the largest retrospective clinical series of extra-visceral, primary retroperitoneal PEComa, leveraging the international collaboration of the Transatlantic and Australasian Retroperitoneal Sarcoma Working Group (TARPSWG).

## Patients and methods

This is an international, multicenter retrospective series of extra-visceral, non-renal, retroperitoneal PEComas diagnosed and treated at 13 sarcoma referral centres, which are part of the Transatlantic Australasian Retroperitoneal Sarcoma Working Group (TARPSWG) between the years 2010 and 2023. Included were adult patients (> 18 years old) with tumors of the PEComa family (PEComa NOS, sclerosing PEComa, and angiomyolipoma) with an extra-visceral, non-renal, primary, soft tissue tumor located in the retroperitoneum, abdomen, or pelvis. Patients with tumors outside the retroperitoneum, abdomen, or pelvis or arising from the retroperitoneal or intraabdominal viscera were excluded.

### Patient Data

The data were extracted from the patient records and included age, sex, personal and family history of malignancy, presentation of the tumor (incidental finding or symptomatic), and imaging characteristics such as type of imaging modality and size and location of the tumor.

### Pathology Details

The data from the pathology report was extracted and analyzed, including tumor size, subtype (PEComa NOS, sclerosing PEComa, or angiomyolipoma), presence of necrosis, mitotic rate (per 10 HPF), Ki-67 index, presence of infiltrative growth, high cellularity, cytological atypia, and immunohistochemical reactivity of tumor cells for Melan A, HMB45, smooth-muscle actin, Desmin, MiTF, and Cathepsin K. Similar data were extracted from the preoperative/diagnostic biopsy report.

### Definitions of the Different Subtypes

Angiomyolipoma was defined as a benign mesenchymal tumor composed of variable proportions of adipose tissue, spindle and epithelioid smooth muscle cells, and thick-walled blood vessels (non-epithelioid AML). PEComa NOS was defined as mesenchymal neoplasms composed of perivascular epithelioid cells (PECs)—distinctive epithelioid cells often closely associated with blood vessel walls and expressing both melanocytic and smooth muscle markers. Epithelioid AML subtypes were included as part of the PEComa NOS, in which > 80% of the tumor is composed of epithelioid cells. Sclerosing subtype of PEComa was defined as subtypes composed of cords and trabeculae of epithelioid cells in a densely collagenous stroma.

### Management of the PEComa

Data were collected regarding treatment intent (curative/non-curative), treatment details—radiation treatment, systemic therapy, surgery, and surgical outcomes—duration of stay, and postoperative complications.

The management and treatment approach included the following modalities:

Radiation therapy: duration, dosage, and whether it was applied in a neoadjuvant, adjuvant, or palliative setting were specified.

Systemic therapy: duration, treatment agent, and whether it was administered as neoadjuvant, adjuvant, or palliative treatment were indicated.

The surgical approach was outlined, including whether the surgery was curative or palliative in intent, the type of resection (simple resection or resection involving adjacent organs), and surgical outcomes such as duration of hospital stay and postoperative complications. Postoperative complications were recorded and classified according to the Clavien–Dindo grading system, which stratifies surgical complications on the basis of the level of intervention required, from grade I (minor deviation from normal postoperative course) to grade V (death of a patient).^10^

Finally, the treatment approach, whether curative or non-curative, was clarified on the basis of the overall therapeutic goal. Patients who were followed alone were considered as non-curative.

### Follow-Up and Survival

All patients were followed up according to local institutional practice, until loss to follow-up or death. Overall survival (OS) was defined as the time interval from the diagnosis to the date of last follow-up or death. Disease-free survival (DFS) was defined as the time interval from the surgical procedure with a curative intent to the date of last follow-up or disease-related recurrence. Disease recurrence was defined by new lesions detected by cross-sectional imaging with/without a biopsy proven of malignancy.

### Statistical Analysis

Data analysis was performed using SPSS version 25 (Armonk, NY) software with a two-sided significance level of *α* = 0.05. Descriptive statistics are presented using prevalence and percentage values for categorical variables, while continuous variables are presented with means and standard deviation; skewed distributed variables are presented by median and range. Comparisons between groups were performed using the chi-squared test or Fisher’s exact test for categorical variables, and the independent samples* t*-test or Mann–Whitney *U* test for continuous variables, as appropriate. Survival analysis was performed using the Kaplan–Meier method with log-rank test for significance.

## Results

**Fig. 1:**
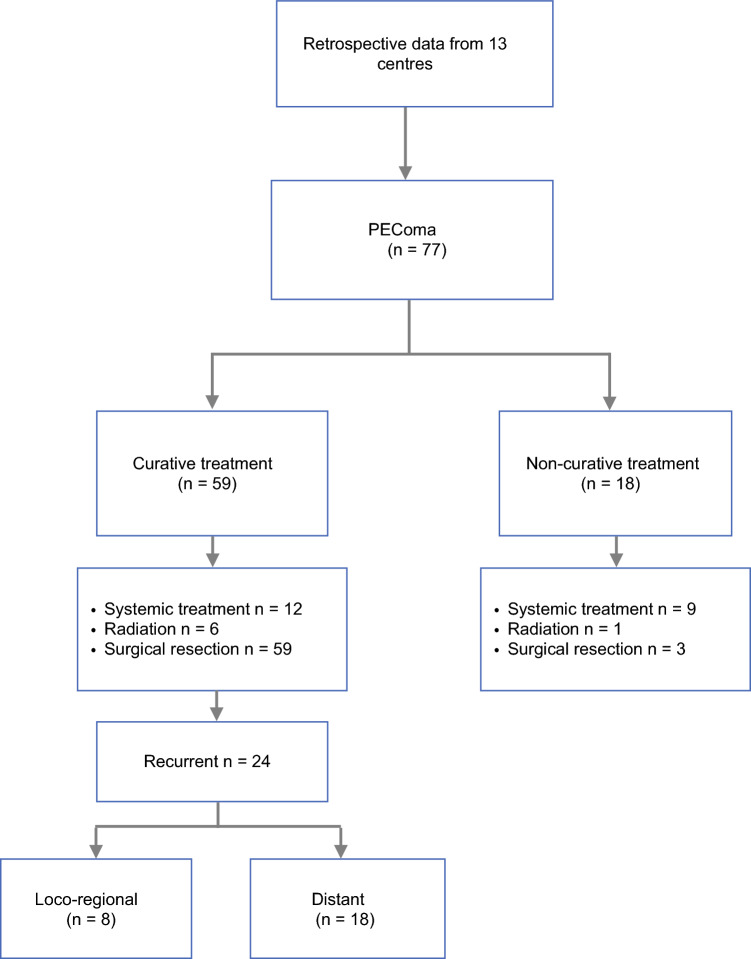
Consort chart

A total of 77 patients from 13 centers were included in the analysis (Consort Fig. 1). The median age of the cohort was 56 years (range 18–81 years) and 73% of patients were female and 51% male. Personal history of tuberous sclerosis was found in two (3%) patients, with no family history of tuberous sclerosis or PEComa family tumors in any of the patients (Table 1).

**Table 1:** Patients’ demographics and characteristics

Variable	PEComa *n* = 77	Curative *n* = 59	Non-curative *n* = 18	p
Median age in years, (range)	56 (18–81)	56 (26–71)	55 (33–81)	
Male,* n* (%)	40 (51%)	14 (23%)	6 (33%)	**0.001**
Female,* n* (%)	37 (49%)	45 (77%)	12 (66%)	
Personal Hx of:				
None,* n* (%)	62 (80%)	51 (86%)	11 (6%)	0.02
Tuberous Sclerosis,* n* (%)	2 (3%)	2 (3%)	0	
PEComa family tumour,* n* (%)	0	0	0	
Unknown,* n* (%)	13 (17%)	6 (10%)	7 (4%)	**0.009**
Family Hx of:				
None,* n* (%)	63 (82%)	52 (88%)	11 (6%)	0.01
Tuberous Sclerosis,* n* (%)	0	0	0	
PEComa family tumour,* n* (%)	0	0	0	
Unknown,* n* (%)	14 (18%)	7 (11%)	7 (4%)	0.01
ASA score, median (range)	1 (1–3)	1(1–3)	1(1–3)	0.4
Presentation:				
Incidental finding,* n* (%)	23 (30%)	15 (25%)	8 (44%)	0.5
Unknown,* n* (%)	2 (3%)	2 (3%)	0	
Symptomatic,* n* (%)	52 (67%)	42 (71%)	10 (55%)	0.4
Pain,* n* (%)	32 (42%)	27 (45%)	5 (27%)	0.3
Gastrointestinal,* n* (%)	10 (13%)	5 (8%)	3 (16%)	0.5
Palpable mass/fullness,* n* (%)	13 (17%)	11 (18%)	1 (5%)	0.4
Organ dysfunction,* n* (%)	5 (6%)	1 (1%)	4 (22%)	**0.008**
Unknown,* n* (%)	2 (3%)	2 (3%)	0	
Imaging:				
CT,* n* (%)	58 (75%)	45 (76%)	13 (72%)	0.9
MRI,* n* (%)	14 (18%)	11 (18%)	3 (16%)	0.9
PET,* n* (%)	2 (3%)	1 (1%)	1 (5%)	0.6
Unknown/missing,* n* (%)	3 (4%)	2 (3%)	1 (5%)	0.9
Location of the tumor				
Left RP,* n* (%)	25 (32%)	16 (27%)	9 (50%)	0.2
Right RP,* n* (%)	17 (22%)	14 (23%)	3 (16%)	0.8
Pelvic,* n* (%)	28 (28%)	25 (42%)	3 (16%)	0.1
Intra-peritoneal,* n* (%)	5 (6%)	2 (3%)	3 (16%)	0.1
Mesenteric,* n* (%)	2 (3%)	2 (3%)	0	
Size on imaging (cm)				
Mean (Sd)	13.9 (19.8)	9.7(5.9)	11.4 (14.1)	
Median (range)	9 (2–25)	9 (1–25)	9 (2–19)	
Biopsy				
None,* n* (%)	18 (23%)	14 (23%)	4 (22%)	0.9
Core,* n* (%)	39 (51%)	29 (49%)	9 (50%)	0.9
FNA,* n* (%)	8 (10%)	4 (7%)	4 (22%)	0.1
Incisional,* n* (%)	4 (5%)	4 (7%)	0	
Excisional,* n* (%)	6 (8%)	4 (7%)	1 (5%)	0.9
Unknown,* n* (%)	2 (3%)	1 (1%)	0	

Bold values are statistically significant, *p* < 0.05

### Presentation

PEComa was initially asymptomatic and identified as an incidental finding on imaging in 23 (30%) patients, while in 52 (67%) it was symptomatic (2; 3% patients unknown). PEComa-related symptoms were pain in 32 (42%) patients, palpable mass or fullness in 13 (17%), gastrointestinal symptoms in 10 (13%), organ dysfunction in 5 (6%), and unknown in 2 (3%).

### Diagnostic Image Characteristics

Images that were used for diagnosis of the PEComa were computed tomography (CT) scan in 58 (75%) patients, magnetic resonance imaging (MRI) in 14 (18%) patients, positron emission tomography (PET) in 2 (3%), and unknown in 2 (3%). The location of the tumor was left retroperitoneum in 25 (32%) patients, pelvic in 28 (28%), right retroperitoneum in 17 (22%), intraperitoneal/mesenteric in 7 (9%). The mean size of the tumor was 13.9 cm (± 19.8) with a median of 9 cm (range 2–25 cm).

### Biopsy and Pathology Subtypes

Preoperative biopsy was carried out in 57 (74%) patients, with 39 (51%) undergoing core biopsy, 8 (10%) fine-needle aspiration, 4 (5%) incisional biopsy, 6 (8%) excisional biopsy, and 2 (3%) unknown.

The tumor subtypes were classified on the basis of final pathology as PEComa not otherwise specified (NOS) in 55 (71%), sclerosing PEComa in 11 (15%), and angiomyolipoma (AML) in 11 (14%). On the basis of the final pathology report, the mean mitotic rate was highest in the PEComa NOS group (13.2/10HPF), followed by sclerosing PEComa (8.5/10 HPF and AML (4/10 HPF). In the PEComa NOS group, infiltrative growth was reported in 14/24 cases (58%), high cellularity in 11/24 cases (46%), and cytologic atypia in 18/24 cases (69%). With immunohistochemistry, there was an expression of HMB45 in 77%, MelanA in 68% of cases, Actin in 65%, and Desmin in 58%. Histopathology characteristics of the whole PEComa cohort based on the different subtypes are summarized in Table 3.

### Biopsy Versus Final Pathology

Of the whole cohort, only 40 (51%) patients had both a preoperative biopsy and a final pathology after complete resection. Pathology was concordant in 36 (90%) patients. Of the remaining, three (7%) had a primary diagnosis of PEComa NOS, while the final pathology was sclerosing type. In one patient, the initial biopsy was PEComa NOS, while the final pathology was AML.

### Management of PEComa

A total of 59 patients (77%) were treated with curative intent and 18 patients with non-curative intent. The reasons for non-curative treatment were metastatic disease in 4 (22%), locally advanced unresectable in 3 (17%), and unknown in 11 (61%); among them were 3 patients with retroperitoneal AML who were managed with surveillance only.

Seven (9%) patients were treated with radiotherapy; six (10%) in the curative group in the adjuvant setting, and one (1%) in the non-curative group as palliative treatment.

In the curative group (*n* = 59), systemic therapy was given to 12 (20%) patients; as chemotherapy (CT) for 6 (10%) patients (2 in the neoadjuvant setting and 4 in the adjuvant setting) and sirolimus to 6 (10%) patients (4 in the neoadjuvant setting and 2 in the adjuvant setting) (Supplementary Table 1). Surgical resection was carried out in 62 (80%) patients, with 59 (76%) resections with curative intent and 3 (3%) with palliative intent (Table 2).

**Table 2:** Management and treatment

Variable	PEComa	Curative	Non-curative	*p*
	*n* = 77	*n * = 59	*n * = 18	
Radiation				
Yes,* n* (%)	7 (9%)	6 (10%)	1 (5%)	
No,* n* (%)	64 (83%)	47 (79%)	17 (95%)	0.5
Missing,* n* (%)	6 (7%)	6 (10%)	0	
Type				
Neoadjuvant,* n* (%)	0	0	0	
Adjuvant,* n* (%)	5 (6%)	5 (8%)	0	
Palliative,* n* (%)	1 (1%)	0	1 (5%)	
Systemic therapy				
Yes,* n* (%)	21 (27%)	12 (20%)	9 (50%)	0.1
No,* n* (%)	56 (72%)	47 (80%)	9 (50%)	0.2
Systemic chemotherapy,* n* (%)	8 (10%)	6 (10%)	2 (11%)	
Targeted therapy (Sirolimus),* n* (%)	12 (15%)	6 (10%)	6 (33%)	
Type				
Neoadjuvant,* n* (%)	6 (7%)	6 (10%)	0	
Adjuvant,* n* (%)	6 (7%)	6 (10%)	0	
Palliative,* n* (%)	9 (18%)	0	9 (50%)	0.001
Surgery				
Yes,* n* (%)	62 (80%)	59 (100%)	3 (16%)	0.00
No,* n* (%)	15 (19%)	0	15 (83%)	1
Intent				
Curative,* n* (%)	59 (76%)	59 (100%)	0	
Palliative,* n* (%)	3 (4%)	0	3 (17%)	
Resection type				
Simple resection,* n* (%)	10 (12%)	9 (15%)	2 (11%)	
Adjacent visceral resection,* n* (%)	49 (63%)	50 (84%)	1 (6%)	0.9
Organs resected				
Colon	10 (12%)	10 (17%)	0	
Rectum	3 (4%)	3 (5%)	0	
Small bowel	2 (2%)	2 (3%)	0	
Kidney	24 (31%)	24 (40%)	0	
Spleen	5 (6%)	5 (8%)	0	
Pancreas	4 (5%)	4 (7%)	0	
Lymph nodes	4 (5%)	3 (5%)	1 (6%)	0.9
Uterus	9 (12%)	8 (13%)	1 (6%)	0.7
BSO	8 (10%)	7 (12%)	1 (6%)	0.8
Bladder	7 (9%)	7 (12%)	0	
Vascular resection/reconstruction,* n* (%)	4 (5%)	4 (7%)	0	
Reasons of not resecting				
Unresectable,* n* (%)	3 (4%)	0	3 (17%)	
Metastatic disease,* n* (%)	4 (5%)	0	4 (22%)	
Comorbidities,* n* (%)	1(1%)	0	1 (6%)	
Benign tumor,* n* (%)	4 (5%)	0	4 (22%)	
Length of stay (days), median (range)	9 (1–56)	8 (1–56)	–	
Post-operative complications (CD)				
Total,* n* (%)	12 (15%)	11 (19%)	1 (6%)	0.5
Severe (CD of ≥ 3),* n* (%)	5 (6%)	4 (7%)	1 (6%)	0.9
I,* n* (%)	2 (2%)	2 (3%)	0	
II,* n* (%)	5 (6%)	5 (8%)	0	
III,* n* (%)	3 (4%)	3 (5%)	0	
IV,* n* (%)	2 (2%)	4 (7%)	1 (6%)	0.7
V,* n* (%)	0	0	0	

### Characteristics of the Curative Intent Group

All of the patients (*n* = 59) in the curative group underwent surgical resection of their tumor: 9 (15%) underwent simple excision of the tumor while 50 (84%) underwent en bloc resection of the tumor with adjacent organs, with kidney (*n* = 24, 40%), colon (*n* = 10, 17%), and uterus (*n* = 8, 13%) as most frequently resected organs. Vascular resection/reconstruction was performed in four (7%) patients. The median length of stay was 8 days (range 1–56 days). The resection status of the curative intent group was R0 in 47 (79%) patients, R1 in 5 (8%), and unknown in 7 (12%). Postoperative complications were observed in 11 patients (19%), including severe complications (Clavien–Dindo grade ≥ III) in 4 patients (7%)

### Outcomes, Disease-Free Survival, and Overall Survival

With a median follow-up of 26 months (2.3–147 months), 24 (40%) of the 59 patients undergoing curative treatment had recurred; 8 (13%) developed loco-regional recurrence, while 18/59 (30%) developed distant metastasis (of them 2 patients had both loco-regional and distant metastasis). Recurrence rates in the curative intent group differed by subtype, with 20 (37%) of the PEComa NOS group, 3 (27%) of the sclerosing PEComa group, and 1 (9%) of the AML group developing recurrence. 17 (22%) patients had died from their disease; 14 (25%) of the PEComa NOS group and 3 (27%) of the sclerosing PEComa group (Table 3). There were 5/9 (55%) recurrences among patients who had simple excision (1 was local and 4 were distant metastases), and 20/50 (33%) in the en bloc organ resection (7 were local and 13 were distant metastases).

**Table 3:** Characteristics based on PEComa subtypes

Variable	PEComa *NOS* *n *= 55	Sclerosing PEComa *n *= 11	Angiomyolipoma *n *= 11	*P* value
Median age in years, (range)	56 (18–81)	47 (26–79)	55 (22–72)	0.6
Male,* n* (%)	12 (21%)	3 (27%)	5 (45%)	0.47
Female,* n* (%)	43 (79%)	8 (73%)	6 (54%)	0.45
Location of the tumor				
Left RP,* n* (%)	16 (29%)	6 (54%)	5 (45%)	0.7
Right RP,* n* (%)	12 (21%)	3 (27%)	1(9%)	0.5
Pelvic,* n* (%)	21 (38%)	2 (18%)	3 (27%)	0.1
Intra-peritoneal,* n* (%)	4 (7%)	0	2 (18%)	0.4
Mesenteric,* n* (%)	2 (3%)	0	0	1
Largest dimensions				
Mean (Sd)	15.1(22.2)	12.6 (7.3)	12.4(5.5)	0.62
Median (range)	8 (1–23)	12 (2–23)	9 (8–21)	
Necrosis % (0-100), n	24	8	5	
Mean (Sd)	44.3 (37.9)	19.6(19.1)	10.4(19.4)	0.7
Median (range)	40 (0–80)	17 (0–50)	2 (0–45)	
Mitotic rate (10/HPF)	29	10	7	
Mean (Sd)	13.2 (14.2)	8.5 (9.8)	4 (7)	0.2
Median (range)	4 (1–74)	3 (0-21)	1 (0–19)	
Ki 67 %	10	6	4	
Mean (Sd)	24.1 (27.7)	4.2(1.7)	14(17)	0.3
Median (range)	11 (1–80)	5 (1–5)	10 (0–19)	
Infiltrative growth,* n* (%)	14/24 (58%)	5/8 (62%)	2/5 (40%)	0.7
High cellularity,* n* (%)	11/24 (45%)	5/9 (55%)	2/5 (40%)	0.8
Cytologic atypia,* n* (%)	18/26 (69%)	8/8 (100%)	5/5 (100%)	0.08
MelanA positive,* n* (%)	17/25 (68%)	4/7 (57%)	5/6 (83%)	0.5
HMB45 positive,* n* (%)	21/29 (72%)	8/8 (100%)	5/7 (71%)	0.23
Actin positive,* n* (%)	7/16 (43%)	8/8 (100%)	5/7 (71%)	0.02
Desmin positive,* n* (%)	11/25 (44%)	8/8 (100%)	3/5 (60%)	0.02
MITF positive,* n* (%)	4/7 (575)	4/4 (100%)	0	0.4
Cathepsin K positive,* n* (%)	4/6 (66%)	2/2 (100%)	0	0.7
Recurrence,* n* (%)	20 (37%)	3 (27%)	1 (9%)	0.2
Death from PEComa,* n* (%)	14 (25%)	3 (27%)	0	0.1

*NOS* not otherwise specificized, *RP* retroperitoneum, *SD* standard deviation

**Fig. 2:**
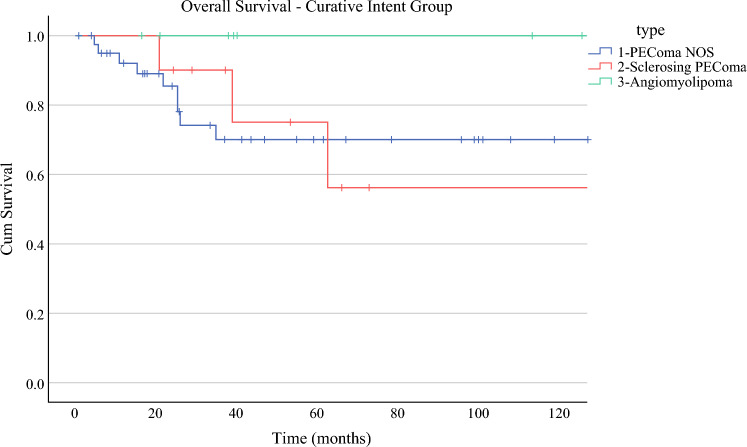
Overall survival based on histology subtype

The estimated 5-year OS of the whole cohort was 63%. The estimated 5-year OS of the curative intent group was 75%, while the non-curative group was 51%. The estimated 5-year OS of the curative group based on histological subtype was 70% for the PEComa NOS group, 75% for the sclerosing PEComa, and 100% for the AML group (Fig. 2).

**Fig. 3:**
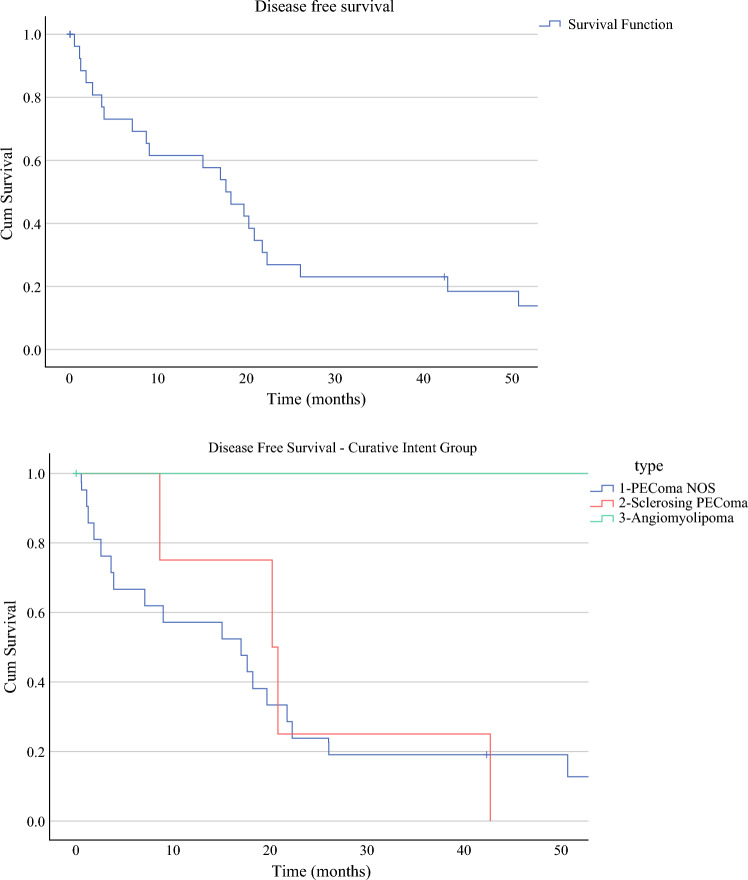
Disease-free survival of the curative group and based on histological subtypes

The estimated 3-year disease-free survival of the curative group was 23%. On the basis of histology, the curative group’s estimated 3-year disease-free survival was 19% for the PEComa NOS group, 25% for the sclerosing PEComa, and 100% for the AML group (Fig. 3).

## Discussion

In this multi-institutional retrospective study, we provide the largest series to date describing the natural history and outcomes of primary extra-visceral retroperitoneal PEComas. Our findings highlight significant differences in clinical behavior between histologic subtypes, with PEComa NOS associated with the highest recurrence and mortality rates, sclerosing PEComa showing an intermediate course, and angiomyolipoma demonstrating predominantly indolent behavior. Importantly, we demonstrate that histological subtype rather than tumor size better predicts oncologic outcomes, and that preoperative biopsy offers a high rate of diagnostic accuracy, supporting its role in treatment planning. These insights have critical implications for prognosis and multidisciplinary management strategies in this rare disease.

Perivascular epithelioid cell tumors (PEComas) are rare mesenchymal tumors composed of epithelioid, or less commonly, spindle cells characterized by unique histological features and immunophenotype, expressing both muscle and melanocytic markers. There appear to be distinct patterns of clinical behavior between various histologic subtypes, with sclerosing PEComa associated with an indolent course and AML usually reflecting benign lesions.^11,12^ In the current series, we saw a low recurrence rate in the AML group with no documented deaths. By contrast, the sclerosing PEComa group reported a 36% recurrence rate and 27% mortality rate. Clinically malignant PEComas usually display high-risk features, such as large tumor size, marked cytological atypia, frequent mitoses, necrosis, and infiltrative growth,^4^ with potential for both local and distant recurrence.^13,14^ Folpe et al. have described 26 cases of PEComa of the soft tissue and gynecological origin and showed an association of size (> 5 cm), high mitotic rate, and necrosis with a more aggressive behavior, with a subclassification of “benign,” “uncertain malignant potential,” or “malignant” based on those worrisome features.^15^ Those criteria have been described in case reports that validate them.^16,17^ Another subtype previously described by Hornick et al. was the sclerosing PEComa, which was identified in the retroperitoneum in 10/13 cases. This subtype demonstrated more diffusely positive SMA and Desmin and limited immunoreactivity for HMB-45. Sclerosing PEComa seems to pursue an indolent course unless associated with a frankly histologically malignant component.^18^ Angiomyolipoma (AML) is a prototypical PEComa subtype defined histologically as a benign mesenchymal neoplasm composed of variable proportions of mature adipose tissue, smooth muscle cells (both epithelioid and spindle), and thick-walled blood vessels.^19^ Reflecting this benign pathology, AMLs typically exhibit an indolent clinical course. They are considered essentially benign lesions, lacking the high-risk histologic features (such as high mitotic activity, marked cytologic atypia, or necrosis) that characterize malignant PEComas.^20^ AML are more frequently found in the kidney and are known as benign lesions that infrequently necessitate intervention. In some cases, during follow-up, there is an increase in size, or they become symptomatic, and an embolization of the lesion may be considered.^21,22^ In the current study, we describe three subtypes in retroperitoneal PEComas. Worrisome features, such as higher mitotic rate and necrosis, are more likely to be seen in PEComa NOS and Sclerosing type, and less likely to be displayed in the AML subtype. The same subtypes have been correlated with oncological outcomes, with the worst prognosis for PEComa NOS, followed by sclerosing PEComa. As expected, AML showed a more indolent and benign behavior. Interestingly, the size of the lesion was similar between the three subtypes, suggesting that size, which is a key component of the sarcoma staging system, is a poor predictor of outcome in patients with PEComa.^23^

Treatment options for retroperitoneal PEComa are also poorly described,^24^ mainly as case reports and small series, with surgical resection as the mainstay of treatment. The largest series, by Hornick et al., describes 6/8 patients undergoing surgical resection with clear margins.^18^ Most other reports are individual case reports with variably aggressive surgical approaches and outcomes.^16,17,25^ In the current series, most tumors (85%) were resected with en bloc resection of adjacent organs, while simple resection was seen in only 12%. This was done safely with a rate of major postoperative complication of 7%, consistent with treatment being offered in high-volume sarcoma centers. Interestingly, although most papers mentioned the lack of consensus in the resection strategies and surgical approach,^14^ most patients who were managed in this series were treated more aggressively, with en bloc resection, compared with a simpler resection described in several case reports.^26–38^ In addition, of all the patients who were treated with curative intent in the current series, 40% developed recurrence, most of them being at distant sites (75% of all recurrences), showing the potential aggressive biology of a subgroup of these tumors, especially in the PEComa NOS and sclerosing variant.

The heterogeneity of retroperitoneal PEComas highlights the critical importance of obtaining preoperative biopsy and achieving an accurate histopathological diagnosis. In our series, there was a 90% concordance between the preoperative biopsy and the final surgical pathology, suggesting that biopsy is generally a reliable diagnostic tool. However, several clinically important considerations must be acknowledged. First, while biopsy can often distinguish between malignant PEComa subtypes and benign lesions such as angiomyolipoma (AML), this distinction is not always straightforward.^39,40^ Overlapping histologic features—such as the presence of epithelioid cells or limited sampling of areas with cytologic atypia—may lead to misclassification. Specifically, there is a risk that a benign AML could be misdiagnosed as a malignant PEComa NOS,^40^ or conversely, that a malignant lesion could be underestimated if high-risk features (e.g., necrosis, high mitotic rate) are missed on limited biopsy samples.^39^ This distinction is critical for clinical decision-making: a malignant diagnosis may lead to aggressive en bloc resections involving adjacent organs, while a benign diagnosis might support more conservative management or active surveillance.^41–44^ Thus, while our data support the use of biopsy in the diagnostic pathway, interpretation should be made in conjunction with imaging, clinical findings, and multidisciplinary discussion, recognizing both the strengths and inherent limitations of biopsy-based diagnosis in these rare tumors.

mTOR inhibitors have transformed the management of malignant PEComas. Accumulating evidence from case series, retrospective analyses, and a pivotal trial demonstrates notable response rates (~40% or higher) and prolonged PFS in patients with advanced or unresectable PEComas treated with agents such as sirolimus, everolimus, or nab-sirolimus.^45,46^ These agents show activity even in bulky retroperitoneal or abdominopelvic tumors, sometimes enabling surgical resection when used neoadjuvantly.^46,47^ Given the rarity of PEComa, formal trials are limited, but all available data consistently support mTOR blockade as an effective therapy. Current guidelines recommend mTOR inhibitor therapy as a potential first-line systemic treatment for advanced PEComas, highlighting its prominent role in achieving disease control in this ultra-rare sarcoma.^45,48^

This study has several limitations that need to be acknowledged. Firstly, being a retrospective study, it is inherently subject to significant selection bias, which may affect the generalizability of the findings. While each site endeavored to identify all consecutive patients who met the inclusion criteria, it is not possible to be confident that cases were not missed; this would particularly be the case for patients with metastatic disease who did not undergo surgical resection. Secondly, there was no centralized pathology review. However, all cases were reported by expert sarcoma pathologists at their home center and concordance of immunohistochemistry was used to confirm subtypes. The study included a small number of cases across various subtypes, each of which received different treatment approaches at different centers, introducing further variability and complexity to the data analysis. Additionally, the retrospective nature of the study led to incomplete data collection, as some patient records were missing or incomplete. This lack of comprehensive data and the small case numbers limited the ability to compare outcomes on the basis of treatments to draw definitive conclusions and create a clear consensus on the most effective treatment strategies. These limitations underscore the need for larger, prospective studies with standardized treatment protocols to better understand and manage this group of rare tumors.

## Conclusions

Retroperitoneal or abdominopelvic PEComa are a rare group of tumors. PEComa NOS and sclerosing PEComa have a more aggressive clinical course than AML. Preoperative biopsy leads to accurate diagnosis in 90% of cases. Patients should be discussed at an expert sarcoma multidisciplinary meeting to optimize the treatment plan.

## Supplementary Information

Below is the link to the electronic supplementary material.

Supplementary file1 (DOCX 17 KB)
