# Correction: Natural History of Primary Retroperitoneal Extra-Visceral Perivascular Epithelioid Cell Tumors (PEC): A Study from Transatlantic and Australasian Retroperitoneal Sarcoma Working Group (TARPSWG)

**DOI:** 10.1245/s10434-025-18021-1

**Published:** 2025-07-29

**Authors:** Eyal Mor, Sameer Apte, Catherine Mitchell, Carolyn Nessim, Max Almond, Bruno Vincenzi, Jose Antonio Gonzalez Lopez, Lee Cranmer, Michael J. Wagner, Aviram Nissan, Miguel Henriques Abreu, Markus Albertsmeier, Mathilda Knoblauch, Adam Barlow, Emily Z. Keung, Giovanni Grignani, Jason L. Hornick, Alessandro Gronchi, David E. Gyorki

**Affiliations:** 1 Sir Peter MacCallum Department of Oncology, Peter MacCallum Cancer Centre, University of Melbourne, Melbourne, VIC Australia; 2 University of Ottawa, Ottawa, Canada; 3 University Hospitals Birmingham NHS Foundation Trust, Birmingham, UK; 4 Università Campus Bio-Medico, Rome, Italy; 5 Hospital de Sant Pau, Barcelona, Spain; 6 University of Washington, Seattle, WA USA; 7 Dana-Farber Cancer Institute, Boston, MA USA; 8 Sheba Medical Center, Ramat-Gan, Israel; 9 Portuguese Institute of Oncology of Porto, Porto, Portugal; 10 Ludwig-Maximilians-Universität (LMU) Munich, LMU University Hospital, Munich, Germany; 11 Leeds Teaching Hospitals NHS Trust, Leeds, UK; 12 The University of Texas MD Anderson, Houston, TX USA; 13 Candiolo Cancer Institute, FPO-IRCCS, Candiolo, TO Italy; 14 Brigham and Women’s Hospital, Harvard Medical School, Boston, MA USA; 15 Fondazione IRCCS Istituto Nazionale dei Tumori, Milan, Italy; 16 Division of Cancer Surgery, Peter MacCallum Cancer Centre, Melbourne, Victoria Australia

**Correction to: Annals of Surgical Oncology**, 10.1245/s10434-025-17787-8

In the original online version of this article, Giovanni Grignani's affiliation was incorrect. The original article was corrected.

