# Supplementary material for: Natural History of Primary Retroperitoneal Extra-Visceral Perivascular Epithelioid Cell Tumors (PEC): A Study from Transatlantic and Australasian Retroperitoneal Sarcoma Working Group (TARPSWG)
Source: Ann Surg Oncol. 2025 Jul 14;32(10):7817–28. doi: 10.1245/s10434-025-17787-8 (PMC12454588; doi:10.1245/s10434-025-17787-8)
Supplement: Supplementary file 1 — Supplementary file1 (DOCX 17 KB) [file 10434_2025_17787_MOESM1_ESM.docx]

Supplementary Table 1 – Treatment and characteristics of the curative groups.

|  | Neoadjuvant  n=6 | Adjuvant  n=6 | Surgery alone  n=47 |
| --- | --- | --- | --- |
| Type of systemic  Chemo, *n* (%)  TKI, *n* (%) | 2 (33%)  4 (66%) | 4 (66%)  2 (33%) | 0  0 |
| Age, median (range) | 52 (31-79) | 47 (35-59) | 57 (18-76) |
| Symptomatic, *n* (%)  Incidental, *n* (%) | 5 (83%)  1(16%) | 5 (83%)  1 (16%) | 33 (70%)  13 (27%) |
| Location:  Right, *n* (%)  Left, *n* (%)  Pelvic, *n* (%)  Intraperitoneal/mesenteric, *n* (%) | 0  3 (50%)  1 (16%)  2 (33%) | 0  0  5 (83%)  1 (16%) | 11 (23%)  14 (29%)  18 (38%)  4 (85) |
| Size on imaging (cm)  Mean (Sd)  Median (range) | 15.9 (6.4)  13(10-25) | 3.4 (1.3)  3 (2.6-5) | 10.1(5.5)  9 (0.6-22) |
| Subtype  PEComa NOS, *n* (%)  Sclerosing PEComa, *n* (%)  Amgiomyolipoma, *n* (%) | 2 (33%)  3 (50%)  1 (16%) | 4 (66%)  1 (16%)  1 (16%) | 35 (74%)  6 (12%)  6 (12%) |
| Resection type  Simple resection, *n* (%)  Adjacent visceral resection, *n* (%) | 0  6 (100%) | 1 (16%)  5 (83%) | 8 (17%)  39 (82%) |
| Post-operative complications (CD)  Total, *n* (%)  Severe (CD of ≥3), *n* (%) | 1 (16%)  0 | 2 (33%)  1 (16%) | 8 (17%)  3 (6%) |
| Recurrence, *n* (%)  Died of disease, *n* (%) | 2 (33%)  1 (16%) | 4 (66%)  1 (16%) | 18 (38%)  9 (19%) |
